# Efficacy and safety of *Gelidium elegans* intake on bowel symptoms in obese adults: A 12-week randomized double-blind placebo-controlled trial

**DOI:** 10.1097/MD.0000000000014981

**Published:** 2019-04-26

**Authors:** Hyoung Il Choi, Jae Myung Cha, In-Kyung Jeong, In-Jin Cho, Jin Young Yoon, Min Seob Kwak, Jung Won Jeon, Soo Jin Kim

**Affiliations:** a Department of Medicine, Graduate School, Kyung Hee University; b Department of Internal Medicine; c Institute of Medical Science, Kyung Hee University Hospital at Gangdong, Seoul, Republic of Korea.

**Keywords:** Defecation, dietary fiber, *Gelidium elegans*, obesity, seaweed

## Abstract

**Background/aims:**

*Gelidium elegans* (*GE*) is known to have antiobesity effects and beneficial effects on functional bowel symptoms in preclinical studies. The aim of this study was to determine the efficacy and safety of *GE* intake on bowel symptoms in obese human adults.

**Methods:**

This 12-week single-center randomized double-blind placebo-controlled study was performed from September 2016 to May 2017. Consecutive obese subjects were randomly assigned (1:1) to either *GE* (1 g) or placebo (1 g) once daily group for 12 weeks. Patients’ bowel symptoms were evaluated using the Bristol Stool Form Scale, Constipation Scoring System (CSS), and Patient Assessment of Constipation-Symptoms (PAC-SYM) questionnaire.

**Results:**

The stool symptom score of PAC-SYM significantly improved in the *GE* group compared with the placebo group after the 12-week treatment (*P* = .041). Abdominal discomfort score of CSS significantly decreased at 12 weeks compared to that at baseline in the *GE* group (*P* = .003), but not in the placebo group (*P* = .398). In addition, abdominal discomfort score of CSS slightly decreased in the *GE* group compared with the placebo group after the 12-week treatment (*P* = .060). However, stool consistency, total CSS score, and PAC-SYM score did not change significantly in both *GE* group and the placebo group over the 12-week treatment period.

**Conclusions:**

*GE* treatment for 12 weeks improved the stool symptom score on the PAC-SYM and abdominal discomfort score on the CSS in obese adults. However, further research is needed in large-scale human studies.

## 1 Introduction

Obesity, one of the rising health concerns worldwide, was recently proven to be associated with various gastrointestinal diseases^[1–3]^ or symptoms.^[4–6]^ Recent studies have shown an increased prevalence of constipation in obese children and a higher prevalence of obesity in children with constipation.^[7–9]^ In a community-based study in Iran, approximately 60% of overweight adults had functional constipation, a higher incidence than that in normal weight individuals of the same community.^[10]^ However, constipation was not reported to be associated with obesity in a recent meta-analysis.^[4]^ Their potential associations may be explained by abnormalities in the motor and sensory activities of the gut in obesity,^[6]^ or shared lifestyle factors such as the consumption of a low-fiber diet or low physical activity levels in obesity.^[11]^

Recent epidemiological and clinical studies have supported the association between constipation and dietary fiber.^[12–15]^ The World Gastroenterology Organization global guideline recommends fiber supplementation for treatment of chronic constipation.^[16]^ However, the American Gastroenterological Association suggested that the association between dietary fiber and constipation does not necessarily indicate causation and the consumption of dietary fiber may not improve bowel function.^[17]^ In a recent National Health and Nutrition Examination Survey, dietary fiber was not a predictor of constipation.^[18]^ In other systematic reviews, based on three randomized controlled trials, soluble fiber was not associated with a reduction in stool consistency or improved successful treatment rate in children with constipation.^[19]^ Therefore, physicians should consider clinical evidence for each dietary fiber regarding bowel movement.^[20]^ In addition, the effect of dietary fiber should be evaluated using an objective measure and validated questionnaire. However, few studies with such a design have been performed to date.

Seaweed has been ingested as a complementary and alternative medicine for constipation in Korea and Japan,^[21]^ but few studies have evaluated its biological function.^[22]^ Recently, *Gelidium elegans* (*G. elegans*), an edible red seaweed native to the intertidal area of the northeastern Asia, was shown to have antiobesity effects in in vitro and experimental studies.^[23–25]^ Furthermore, *G. elegans* had a positive effect on functional bowel symptoms and bowel movement in another experimental study.^[26]^ However, no evidence has yet been found to support its effect on bowel symptoms and bowel movement in obese adults. Therefore, this study aimed to determine the efficacy and safety of *G. elegans* on bowel symptoms and bowel movement in obese human adults.

## 2 Materials and methods

### 2.1 Study population

Subjects were recruited by advertisement on public notice boards and the homepage of Kyung Hee University Hospital at Gangdong. Consecutive subjects with a body mass index (BMI) of 25–30 kg/m^2^ who were aged ≥ 20 years were recruited for this study, as obesity is defined as a BMI ≥ 25 kg/m^2^ in the Asian population.^[27]^ Subjects were excluded, if they had taken gastrointestinal medication (antispasmodic, laxative, or antidiarrheic drugs), weight-reducing medication, statins, or glucocorticoids within 12 weeks before the screening visit to rule out secondary obesity; had intake of dietary supplements potentially interfering with this trial (probiotics, prebiotics, or supplements rich in fiber); had neurological or psychiatric disorders including depression; were vegetarian or vegan; were pregnant or breastfeeding; abused alcohol or drugs; or had significant laboratory abnormalities (serum transaminase ≥ 3 times the normal limits, serum creatinine ≥ 1.5 mg/dL, or thyroid-stimulating hormone [TSH] ≥ 10 mIU/L). Individuals who were unable to provide informed consent or were in poor general condition (American Society of Anesthesiologists grade > III) were also excluded. Women of child-bearing potential were required to ensure adequate contraceptive protection during the study.

### 2.2 Study design and efficacy

**Figure 1:**
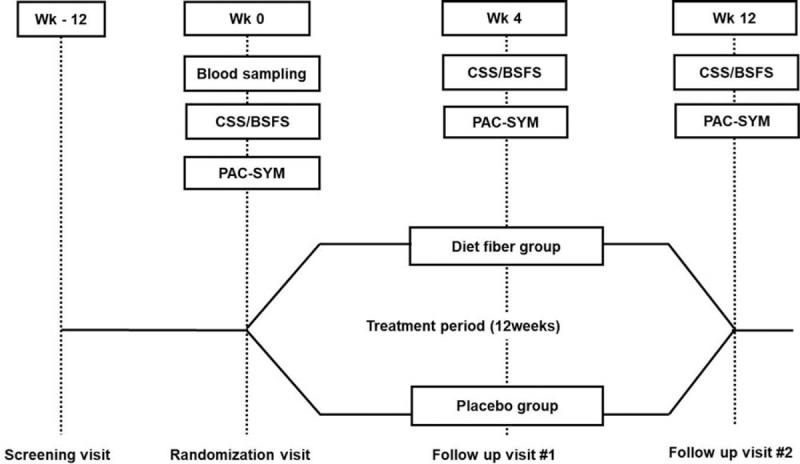
Study design. This 12-week single-center randomized double-blind placebo-controlled study consisted of a screening visit followed by 12-week blinded consumption of the study product. At the second visit, subjects were randomly assigned (1:1) to the *Gelidium elegans* group or the placebo group. BSFS = Bristol stool form scale, CSS = constipation scoring system, PAC-SYM = Patient Assessment of Constipation-Symptoms.

This 12-week single-center randomized double-blind placebo-controlled study was conducted from September 2016 to May 2017. The study consisted of a screening visit followed by a 12-week blinded consumption of the study product (Fig. 1). As most clinical trials with dietary fiber were performed for 4 weeks up to 12 weeks in previous studies,^[28,29]^ a 12-week trial was adopted in this study. After providing written informed consent, subjects were screened against the inclusion and exclusion criteria, were informed about the data collection, and voluntarily participated in this study. A detailed list of diet or foods especially rich in fiber was provided to the subjects at the screening visit. At the second visit, consecutive subjects were randomly assigned (simple randomization with allocation ratio 1:1) to either *G. elegans* or placebo group according to a computer-generated randomization schedule. Randomized assignments numbers were placed in a sealed envelope and opened by the coordinator nurses for randomization. Eligible subjects received their study product from the hospital pharmacist involved in this study. The subjects and researchers involved in this study were blinded to the interventions until the final database unlock. The primary outcome was the change in the subject's bowel symptoms, whereas the secondary outcome was the difference in the Patient Assessment of Constipation-Symptoms (PAC-SYM) scores between the *G. elegans* and placebo groups.

### 2.3 Data collection

Vital signs, clinical laboratory tests (complete blood count, chemical test, lipid profiles, and TSH level), urine human chorionic gonadotropin test, and physical examinations were performed at the screening visit and repeated routinely at 4 and 12 weeks. During the study period, patients recorded their study product intake, adverse events (AEs), and diet details in a daily diary. As the Common Terminology Criteria for AEs has been widely used as the predominant set of toxicity criteria for cancer clinical trials and scientific meetings,^[28]^ moderate (grade 2) changes at any time after the first intake of study product was defined as a significant laboratory AEs: that is, hypoglycemia (<55 mg/dL), hypercholesterolemia (>300 mg/dL), elevated transaminase (>2.5 times the upper normal limit), azotemia (>1.3–1.8 the upper normal limit), leukopenia (<3.0 × 10^3^/mm^3^) or leukocytosis (>15.0 × 10^3^/mm^3^), and anemia (<10.0–8.0 g/dL).^[30]^

Questionnaires about bowel symptoms were completed at the screening visit and repeated at 4 and 12 weeks. The consistency of each bowel movement was classified using the Bristol Stool Form Scale (BSFS).^[31]^ A global assessment of each patient's bowel movements was retrospectively performed over 4 weeks and 12 weeks using the Constipation Scoring System (CSS), which was based on eight variables (frequency of bowel movements; difficult or painful evacuation; completeness of evacuation; abdominal pain; time per attempt; type of assistance, including laxatives, digitations, or enemas; number of unsuccessful attempts at evacuation in a 24-h period; and duration of constipation).^[32,33]^ Seven CSS items were scored using a five-point Likert scale of 0 (none of the time) to 4 (all the time), whereas one CSS item was rated on a 0 to 2 scale. A total score can range from 0 (normal) to 30 (severe constipation). Additionally, patient symptoms were measured using the PAC-SYM questionnaire, which is frequently used in clinical trials of constipation.^[34]^ On the PAC-SYM, the severity of 12 symptoms was scored on a five-point Likert scale (0 = “symptom absent,” 1 = “ mild,” 2 = “ moderate,” 3 = “ severe,” and 4 = “ very severe”) and the items were grouped into three subscales: stool, abdominal, and rectal symptoms. A mean total score in the range of 0 to 4 is generated by dividing the total score by the number of questions completed. Higher score reflects greater severity.

### 2.4 Study products and compliance

Study products were 1 g/day *G. elegans* or 1 g/day placebo (primarily consisting of microcrystalline cellulose). The study products were provided by Naturetech Corp. (Jincheon-gun, Chungcheongbuk-do, Republic of Korea), which has pioneered Korea's dietary supplements industry since 1976. Simply, the *G. elegans* product was washed with water to remove the salt; it was first extracted with 70% alcohol and then extracted with hot water, filtered, concentrated, and packed after spray drying. Study product is managed according to the manufacturing guidelines of “Korean Ministry of Food and Drug Safety”“ by a self-quality test and authorized quality test, especially in the areas such as bacterial or fungal contamination. Study products were delivered as opaque sachets containing six study product tablets labeled with the respective subject number.

As *G. elegans* has been shown to exhibit antiobesity effects^[23–25]^ and have a beneficial effect on bowel symptoms,^[21,26]^ it was selected as a potential candidate for bowel symptoms in obese adults. Microcrystalline cellulose was selected as the placebo because its taste and appearance resemble those of *G. elegans*; moreover, it is the most commonly used excipient for tablets. The study product tablets were consumed once a day (after breakfast). The time of the study product intake was documented in the subject's diary. All unused sachets had to be returned to the study site in order to calculate the patient compliance. During the study, the subjects were not allowed to change their normal dietary or physical activity habits by recording a diary for their diet and physical activity during the study periods. Furthermore, participants were instructed to have a regular diet and regular physical activities and provided dietary information, including the components of a balanced diet, the importance of food choice, and instructions on cooking methods.

### 2.5 Ethics

The trial was performed in accordance with the Declaration of Helsinki, International Conference on Harmonization-Good Clinical Practice guidelines, and local laws and regulations. The study protocol was reviewed and approved by our hospital's Institutional Review Board (KHNMC 2016-05-039). This study was retrospectively registered and approved in www.cris.nih.go.kr, which is a nonprofit online registration system for clinical trials to be conducted in Korea (KCT0003514). Signed written informed consent was obtained from all subjects at their first visit.

### 2.6 Statistical analysis

The present study was planned as an efficacy test; therefore, the principle analysis was performed on the per-protocol population. However, all randomized subjects who took at least one dose of the study product were included in the safety analysis (intention-to-treat). The sample size estimation was based on an expected 30% difference in the subject's symptoms between the *G. elegans* and placebo groups. For a two-sided significance level of 0.05 and a power of 0.8, a sample size of 39 was calculated with the formula n = 2(*Z*_*α*/2_ + *Z*_*β*_)^2^*σd*^2^/(*μ*_d*t*_ − *μ*_d*c*_)^2^.

Unless indicated otherwise, the results are expressed as number (percentage) or median (interquartile range [IQR]). For the parametric analysis, the *t*-test or analysis of variance test was used for continuous variables, while the *χ*^2^ test was used for categorical variables. For nonparametric analyses, the Wilcoxon signed-rank test was used. Normality was assessed using the Shapiro–Wilk test. The analysis of covariance method was used to detect intergroup differences in the changes or differences in each visit time relative to the baseline measurements. Additionally, to use the analysis of covariance method, the residual plot was checked and a normality test was performed. The difference in visit time in each group was analyzed using the paired *t*-test method. Two-sided *P* values <.05 were considered statistically significant. All statistical analyses were performed with the Statistical Package for the Social Sciences version 18.0 (SPSS Inc., Chicago, IL, USA).

## 3 Results

### 3.1 Patient demographics and disposition

**Figure 2:**
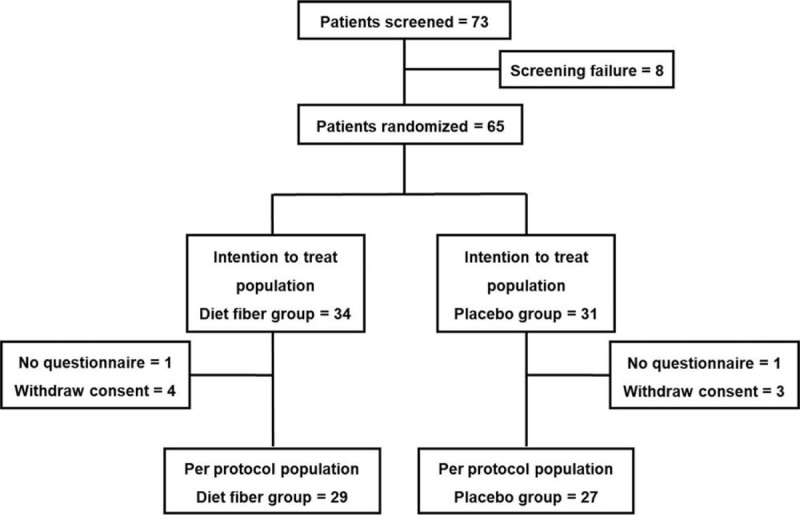
Flow chart of subjects. During the study period, 73 patients were screened; of them, 65 were randomized to treatment with *Gelidium elegans* or placebo. Of the 65 randomized patients, 58 completed the 12-week study and diary (*G. elegans* group = 27 vs. placebo group = 29). Four and five subjects dropped out of the placebo and dietary fiber groups, respectively.

Figure 2 summarizes the trial flow, number of patients in each group, and number of (and reasons for) withdrawals. During the study period, 73 patients were screened, of which 65 were randomized to either the *G. elegans* treatment group (n = 34) or placebo group (n = 31). Of the 65 randomized patients, 58 completed the study and the 12-week diary (*G. elegans* group = 27 vs. placebo group = 29). Four and five subjects dropped out of the placebo and *G. elegans* groups, respectively.

Demographic and baseline laboratory data for both groups are shown in Table 1. No significant intergroup differences in age, sex, BMI, or laboratory data were found. In the assessment of the baseline characteristics of the bowel movements, stool consistency measured by the BSFS, CSS score, and PAC-SYM score did not differ between the two groups (Table 2).

**Table 1:**
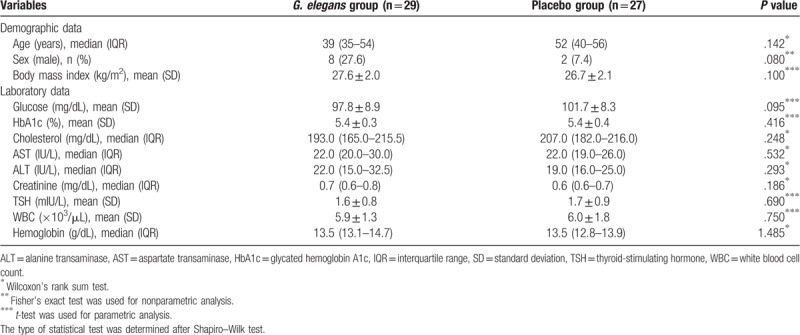
Baseline demographic and laboratory characteristics of study participants.

**Table 2:**
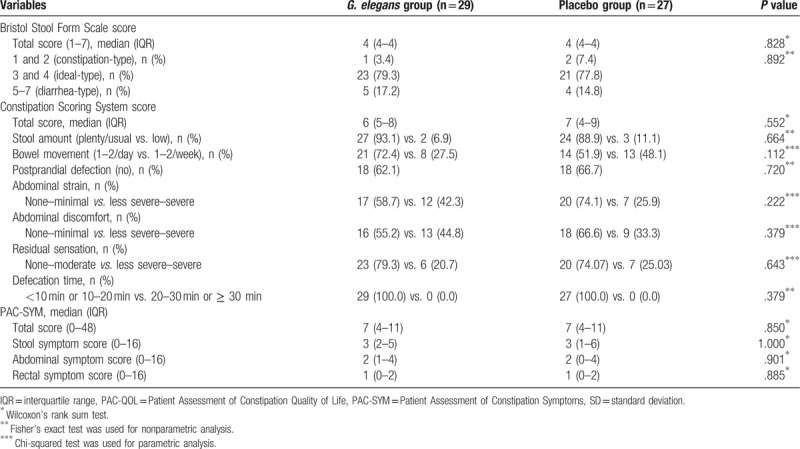
Baseline characteristics of the bowel movement of study participants.

### 3.2 Efficacy analysis

Over the 12-week treatment period, stool consistency and patient symptoms were measured using the BSFS, CSS, and PAC-SYM. When the variation on the BSFS and CSS was compared at the end of the treatment, no difference was found between the dietary fiber and placebo group (*P* > .1 for both). Patients’ bowel symptoms did not differ significantly at the end of the treatment (Tables 3 and 4). However, the stool symptom score of the PAC-SYM significantly decreased in the dietary fiber group compared to the placebo group after the 12-week treatment (*P* = .041). The abdominal discomfort score on the CSS score significantly decreased at 12 weeks compared to the baseline in the dietary fiber group (*P* = .003), but not in the placebo group (*P* = .398). In addition, abdominal discomfort score on the CSS slightly decreased in the dietary fiber group compared to the placebo group after the 12-week treatment (*P* = .060).

**Table 3:**
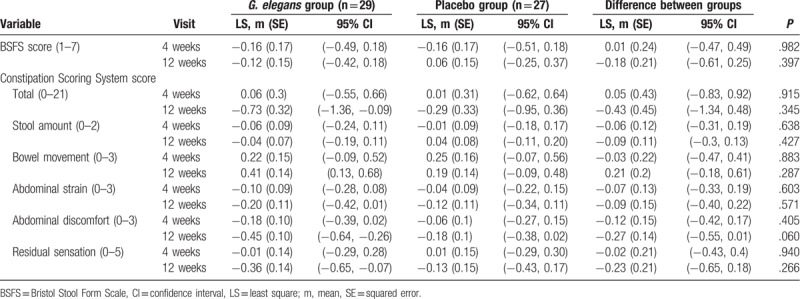
Changes in bowel movement and symptoms from baseline associated with treatment over the 12-week treatment period.

**Table 4:**
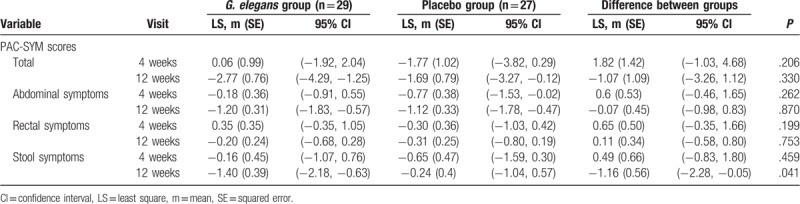
Patient Assessment of Constipation Symptoms (PAC-SYM) scores in the dietary fiber and placebo groups over the 12-week treatment.

### 3.3 Safety and tolerability

The safety and tolerability profiles were excellent, as no treatment-related AEs, serious AEs, or cases of treatment discontinuation were reported in either group. No clinically relevant changes in vital signs or laboratory parameters measured in this study were detected over time. Furthermore, no important differences in the incidence of treatment-emergent laboratory abnormalities were noted in either group.

## 4 Discussion

This is the first 12-week randomized double-blind placebo-controlled study to evaluate the efficacy and safety of *G. elegans* intake on bowel symptoms in obese human adults, using an objective measurement tool for stool consistency and validated questionnaires for bowel symptoms (BSFS, CSS, and PAC-SYM). It is noteworthy to point out that the beneficial effect of dietary fiber on bowel symptoms is still a complex issue that remains to be confirmed with objective and validated measurement tools. In our study, the stool symptom score on the PAC-SYM significantly decreased in the dietary fiber group compared to the placebo group after the 12-week treatment. The abdominal discomfort score on the CSS significantly decreased at 12 weeks compared to the baseline in the dietary fiber group, but not in the placebo group. In addition, abdominal discomfort score on the CSS slightly decreased in the dietary fiber group compared to the placebo group after the 12-week treatment. Even though our findings need further follow-up research, *G. elegans* intake may be recommended as the optimal treatment option for fiber consumption in obese adults.

Dietary fiber is defined as nondigestible carbohydrates and lignin that are intrinsic and intact in plants.^[35]^ Epidemiological studies have supported the association but not the causation between constipation and dietary fiber.^[12–15]^ Dietary fiber was not a predictor of constipation in a recent population-based study,^[18]^ and soluble fiber failed to reduce the stool consistency measured by the BSFS.^[19]^ The inconsistencies in the current body of knowledge may highlight the importance of dietary fiber type and study design. To enable the laxative effect of dietary fiber, a fiber must (1) resist fermentation to remain intact in the large bowel and stool and (2) significantly increase the stool water content and stool output.^[20]^ The laxative effect of dietary fiber is likely to be based on the chemical structure of the fiber, which varies in the chain length, branching, side chains, type of binding, and composition.^[36]^ In our study, *G. elegans* was selected as a potential candidate for bowel symptoms in obese adults as it has been used as a complementary and alternative medicine for constipation^[21]^ and has been shown to exhibit antiobesity effects in vitro and experimental studies.^[23–25]^

Korea, Japan, and parts of China consume the greatest proportion of the 2 billion kg of seaweed harvested each year for human consumption.^[37]^ According to the epidemiological studies comparing Asian and Western diets, the consumption of seaweed has a beneficial effect on some chronic diseases such as cancer, hyperlipidemia, and coronary heart disease.^[38–40]^ Furthermore, certain seaweed fibers have beneficial effects on gut health^[41,42]^ and potential prebiotic activity.^[43,44]^ Seaweed fiber appears to have different chemical, physicochemical, and fermentation characteristics from those of higher-order plant carbohydrates, but very little is known about its beneficial effects in the human gut health.^[43]^ According to an *in vitro* study performed by Ramnani et al,^[44]^*Gelidium* extract treatment induced a significant increase in the number of bifidobacterial populations in human feces. Furthermore, the fermentation of *Gelidium* extracts produced the highest production of total short-chain fatty acids and acetic and propionic acids among several examined seaweed extracts. The prebiotic activity of *Gelidium* extracts may explain its beneficial effect on bowel symptoms seen in our study. Therefore, our study may become a pilot study on this issue, and further studies can increase our understanding of the potential role of *G. elegans* in gut health.

Our study is the first human study on the beneficial effect of *G. elegans* on bowel symptoms. However, this study has some limitations that warrant consideration. First, it was a single-center study based on a small sample size, limiting the generalizability of our findings. However, our study has an excellent design and it may offer a considerable body of evidence to aid future studies examining the efficacy of *G. elegans* intake. Considering only three (5.4%) patients had a BSFS score <3, its effect should be reevaluated in patients with constipation. Second, it was difficult to assume the optimal dose of seaweed fiber in our study since no previous study has examined this issue. As *G. elegans* intake at 50 mg/kg/day and 200 mg/kg/day had a beneficial effect on the lipid profile compared to the placebo in a previous rat model,^[45]^ the human equivalent dose was calculated as 16.2 mg/kg (approximately 1.0 g for a 65-kg adult) based on dose translation from animal to human studies.^[46]^ Third, the generalizability of our findings may be limited to normal weight subjects as only obese patients were included in this study. Because the BMI has been found to be inversely associated with constipation^[47]^ and the antiobesity effects of *G. elegans* have been reported,^[23–25]^ obese adults were considered a potential target group in our study. Fourth, it needs to be mentioned that we did not collect the stool sample to check the amount and consistency of stool; however, it may be a noncompliance design. Finally, we failed to enroll enough participants to match the calculated sample size, and there was a difference of three cases between the experimental group and the control group because the block size was not specified when the random number was generated. Our trial was retrospectively registered as we omitted a prospective registration since our study was a small pilot study using red seaweed, which is commonly consumed in Korea.

In conclusion, 12-week *G. elegans* treatment improved the stool symptom score on the PAC-SYM and decreased abdominal discomfort score on the CSS in obese adults. However, the efficacy and mechanism of *G. elegans* on the gut health should be further clarified in large-scale human studies.

## Acknowledgments

This work was supported by the Industry Technology Development Program of Fisheries Food, 20150349, Development of functional ingredient approved by MFDS and global finished product for improving metabolic syndrome using *Gelidium elegans* by the Ministry of Oceans and Fisheries, Republic of Korea.

## Author contributions

**Conceptualization:** Hyoung Il Choi, Jae Myung Cha, Min Seob Kwak.

**Data curation:** In-Kyung Jeong, In-Jin Cho, Min Seob Kwak.

**Formal analysis:** Jae Myung Cha, In-Kyung Jeong, In-Jin Cho, Jin Young Yoon, Min Seob Kwak, Jung Won Jeon, Soo Jin Kim.

**Funding acquisition:** In-Kyung Jeong.

**Investigation:** Min Seob Kwak.

**Methodology:** Jae Myung Cha, Soo Jin Kim.

**Project administration:** In-Jin Cho.

**Resources:** In-Kyung Jeong, Jin Young Yoon.

**Software:** In-Jin Cho, Soo Jin Kim.

**Supervision:** Jin Young Yoon, Jung Won Jeon.

**Validation:** Jae Myung Cha, Jung Won Jeon.

**Writing – original draft:** Hyoung Il Choi, Jae Myung Cha.

**Writing – review & editing:** Jae Myung Cha.
